# Supplementary material for: Adaptation of a Model Spike Aptamer for Isothermal Amplification-Based Sensing
Source: Sensors (Basel). 2024 Oct 26;24(21):6875. doi: 10.3390/s24216875 (PMC11548252; doi:10.3390/s24216875)
Supplement: Supplementary file 1 [file sensors-24-06875-s001.zip › sensors-3243082-supplementary.pdf]

Supplementary material for:

Adaptation of a Model Spike Aptamer for Isothermal Amplification-Based Sensing

Emre Yurdusev, Pierre-Luc Trahan, Jonathan Perreault

INRS-Centre Armand-Frappier Santé Biotechnologie, Laval, Québec, Canada H7V 1B7.

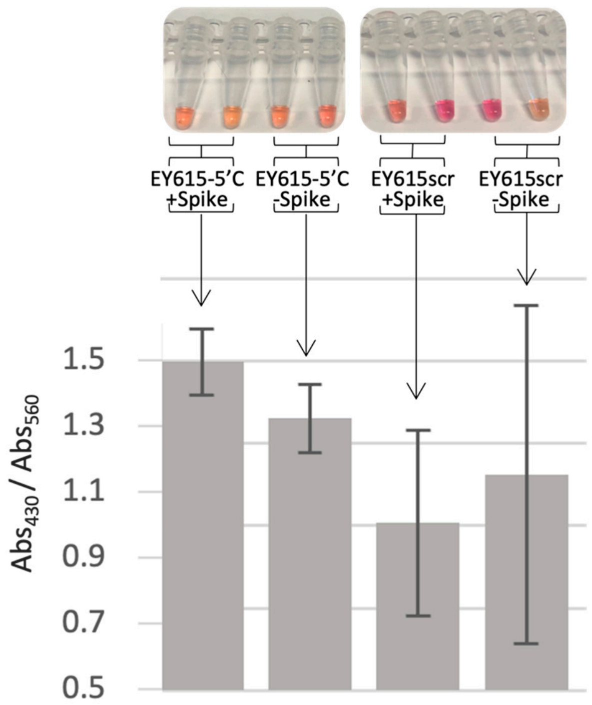

Figure S1: LAMP tests results with EY615-5'C and EY615scr designs

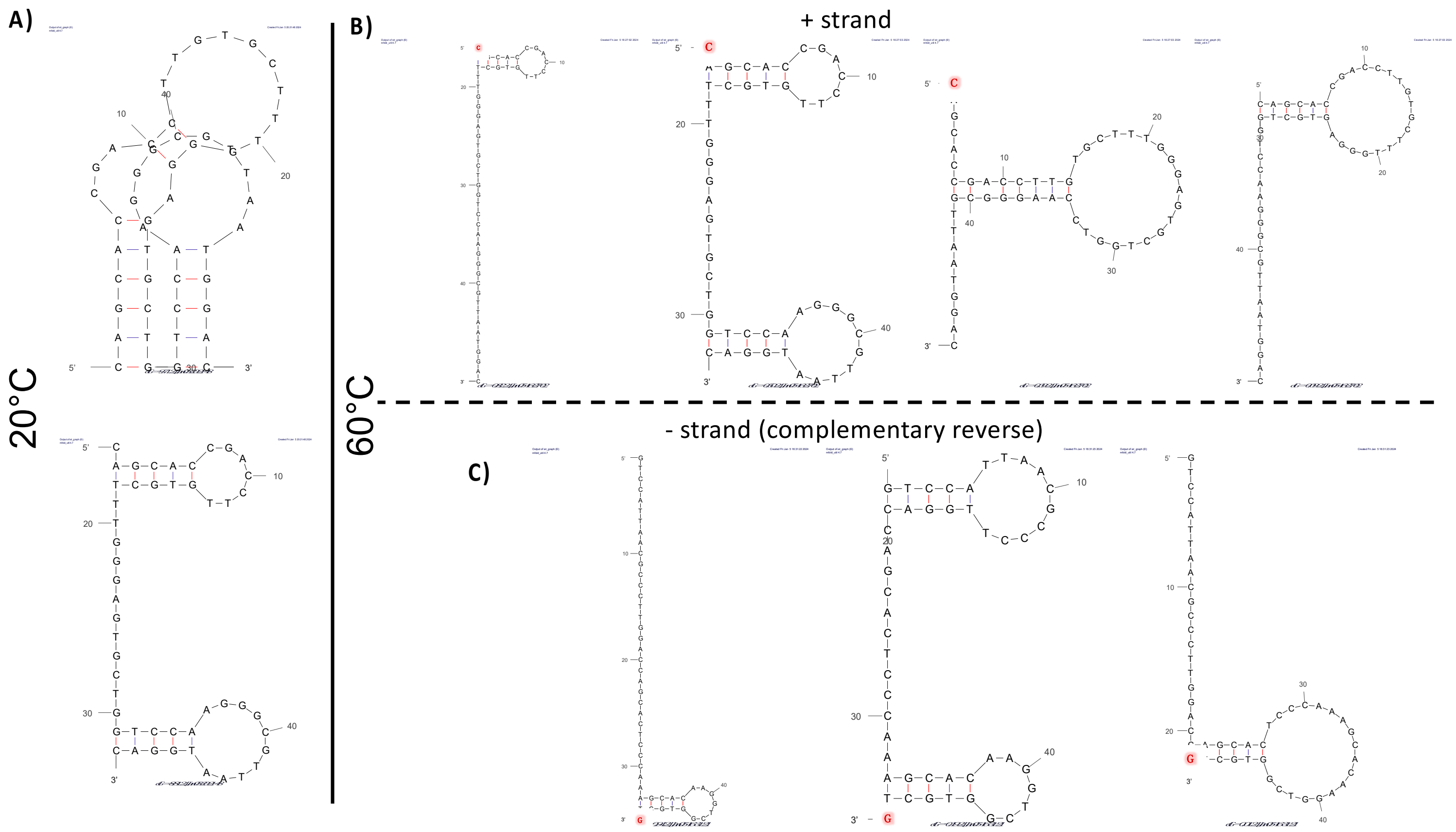

Figure S2: Complete Structural Predictions by UNAFold[1]: Temperature-Dependent Analysis of Spike-Aptamer and Its Complementary Sequence under RCA and LAMP Conditions. A) Structure predictions at 20°C, the temperature at which RCA reactions are initiated. B) and C) Structure predictions at 60°C, the operational temperature for LAMP reactions. In B), all predicted structures of the EY615 aptamer (referred to as the + strand) are shown. C) illustrates the structures for the complementary reverse sequence of EY615 (- strand), which is also crucial in the LAMP process (refer to Fig.1). For the predictions at 60°C, the nucleotide removed in the EY615-5'C design is highlighted in red. This highlights that in certain predicted structures, this nucleotide appears problematic for LAMP-compatible configurations.

Name	Notes	Sequence
EY615 *	Main aptamer design	P-CAGCACCGACCTTGTGCTTTGGGAGTGCTGGTCCAAGGGCGTTAATGGAC
EY615-5'C *	Aptamer design with C in 5' lacking	P-AGCACCGACCTTGTGCTTTGGGAGTGCTGGTCCAAGGGCGTTAATGGAC
EY615scr *	Scrambled ctrl sequence	GCCCAACTCGACTGTCGGTTAGGTTTGGGCGCACTCGAGTAGTAGAGTGC
EY615rdl *	Design with reinforced dumbbell-like shape (ctrl + for LAMP)	CAGCACGCACATTATAGTGCGTGCTGATATTGTCCAAGACGGATGTTCGTCTTGGAC
EY615lv *	Long version aptamer (ctrl – for LAMP, see Song *et al.*[2])	TATCCAGAGTACGCAGCACCGACCTTGTGCTTTGGGAGTGCTGGTCCAAGGGCGTTAATGGACACGGTGGCTTAGTA
EY616	Forward primer to EY615 (LAMP compatible primers, LCP)	CAGCACCGACCTTGTGCTTTG
EY617	Reverse primer to EY615 (LAMP compatible primers, LCP)	GTCCATTAACGCCCTTGGACC
PLT098	Forward primer to EY615scr	GCCCAACTCGACTGTCGGTTA
PLT099	Reverse primer to EY615scr	GCACTCTACTACTCGAGTGCG
EY637	Reverse primer to EY615rdl	GTCCAAGACGAACATCCGTC
EY636	Forward primer to EY615rdl	CAGCACGCACATTATAGTGC
EY631	Forward primer to EY615-5'C (Reverse primer is EY637, same as EY615)	AGCACCGACCTTGTGCTTTGG
EY632	Forward primer to EY615lv	TATCCAGAGTACG CAGCACCG
EY633	Reverse primer to EY615lv	TACTAAGCCACCGTGTCCATTAACG
EY641	Forward primer to EY615 (HRCA specific, HSP, see Fig. S3 for details)	CTCCCAAAGCACAAGGTCGG
EY642	Reverse primer to EY615 (HRCA specific, HSP, see Fig. S3 for details)	GCTGGTCCAAGGGCGTTAA
EY615QF	EY615 with fluorescent and quencher conjugates	Cy5/CAGCACCGACCTTGTGCTTTGGGAGTGCTGGTCCAAGGGCGTTAATGGAC/BHQ2
EY620	Partial reverse complementary to EY615 (for EY615QF tests)	TTAACGCCCTTGGACCAGCACTCCCAAAGCACAAGGTCGG

Conformational Structures of Key Sequences (marked with an asterisk in the list above)

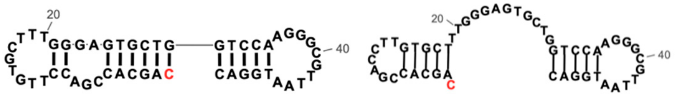

EY615 & EY615-5'C (with C in red lacking)

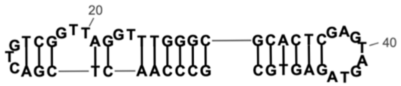

EY615scr

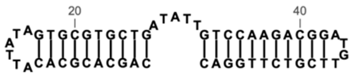

EY615rdl

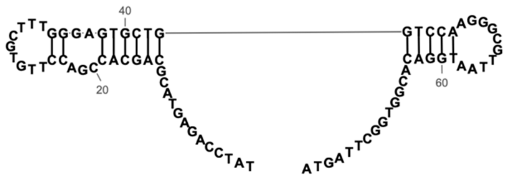

EY615lv

Table S1: Comprehensive List of Primer, Aptamer, and Control Oligonucleotide Sequences.

RCA/LAMP colorimetric assays (Tecan Infinite® M1000 plate reader data)

	LAMP									RCA			
			supplementary assays & ctrls							LAMP compatible primers		RCA specific primers	
	EY615		EY615-5'C			EY615scr		EY615lv	EY615rdl	EY615		EY615	
Abs430	+ Spike	- Spike	+ Spike	- Spike	+ Cell.B	+ Spike	- Spike			+ Spike	- Spike	+ Spike	- Spike
duplicate 1	0.53	0.42	0.52	0.45	0.48	0.49	0.43	0.38	0.60	0.53	0.52	0.35	0.47
duplicate 2	0.56	0.42	0.54	0.49	0.49	0.43	0.51	0.41	0.55	0.52	0.53	0.40	0.56
Abs430													
duplicate 1	0.40	0.56	0.42	0.37	0.48	0.46	0.58	0.55	0.42	0.29	0.27	0.50	0.24
duplicate 2	0.41	0.56	0.39	0.45	0.48	0.58	0.39	0.45	0.37	0.27	0.28	0.47	0.31
**Ratio 430/560**													
duplicate 1	1.34	0.74	1.24	1.22	1.00	1.07	0.73	0.70	1.41	1.79	1.91	0.71	1.95
duplicate 2	1.37	0.75	1.36	1.10	1.04	0.75	1.32	0.91	1.49	1.89	1.94	0.84	1.81
**Average**	**1.36**	**0.75**	**1.30**	**1.16**	**1.02**	**0.91**	**1.02**	**0.80**	**1.45**	**1.84**	**1.92**	**0.77**	**1.88**
Std. dev.	0.02	0.00	0.08	0.08	0.02	0.23	0.41	0.15	0.06	0.07	0.02	0.09	0.10

Data used in **Fig. 2c** (Average row)

Mechanistic study / Fluorometric assays with EY615QF design (Typhoon FLA 9500 scanner data)

EY615QF + complementary oligonucleotide (EY620) assays

[EY615QF]	+ EY620	- EY620
100nM	8.36E+07	1.83E+06
10nM	3.81E+05	1.50E+05
1nM	4.57E+04	2.39E+04

Assay's buffer background fluorescence subtracted

EY615QF + Spike and primers (EY617 & EY616) assays

	+ primers		- primers	
	+ Spike	- Spike	+ Spike	- Spike
duplicate 1	4.27E+06	3.30E+06	5.23E+05	3.61E+05
duplicate 2	3.89E+06	2.91E+06	4.80E+05	3.25E+05
Average	4.08E+06	3.11E+06	5.01E+05	3.43E+05
Standart dev.	2.67E+05	2.72E+05	3.06E+04	2.58E+04

Data used to calculate fluorescence switch values (ratios*)

	+ Spike		- Spike		Ratio*
+ Primers	4.08E+06	+/- 2.67E+05	3.11E+06	+/- 2.72E+05	**1.31**
- Primers	5.01E+05	+/- 3.06E+04	3.43E+05	+/- 2.58E+04	**1.46**
**Ratio***	**8.14**		**9.05**		

*: Fluorescence value of the assay with the variable element / Fluorescence value of the assay without the variable element

Table S2: Raw data of Colorimetric Analysis (from Tecan Infinite® M1000) and Fluorescence Measurement (from GE Typhoon FLA 9500 scanner)

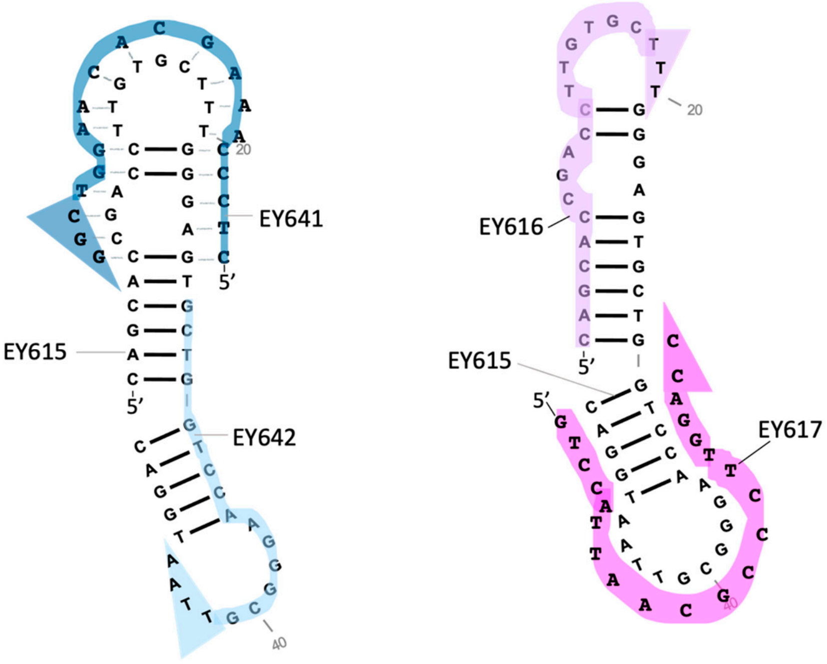

Figure S3: EY615 and LAMP Compatible Primers (LCP), EY616 & EY617, and HRCA Specific Primers (HSP), EY641 & EY642. This figure presents the sequences of the aptamer EY615 alongside the binding sites for the LCP (left) and HSP (right) primers pair. A triangle at the end of the primer shape represents the direction of amplification that would be initiated by the primers. For experimental setup with HSP, in cases where EY615 does not undergo self-ligation, the resulting amplification product is notably shorter. This shortened product lacks adequate binding sites for EY642, as depicted in the figure. The absence of suitable binding sites for EY642 on these shorter amplicons implies that Loop-Mediated Isothermal Amplification (LAMP) is unlikely to proceed effectively with these specific amplification products, as EY642 is essential for initiating the LAMP process.

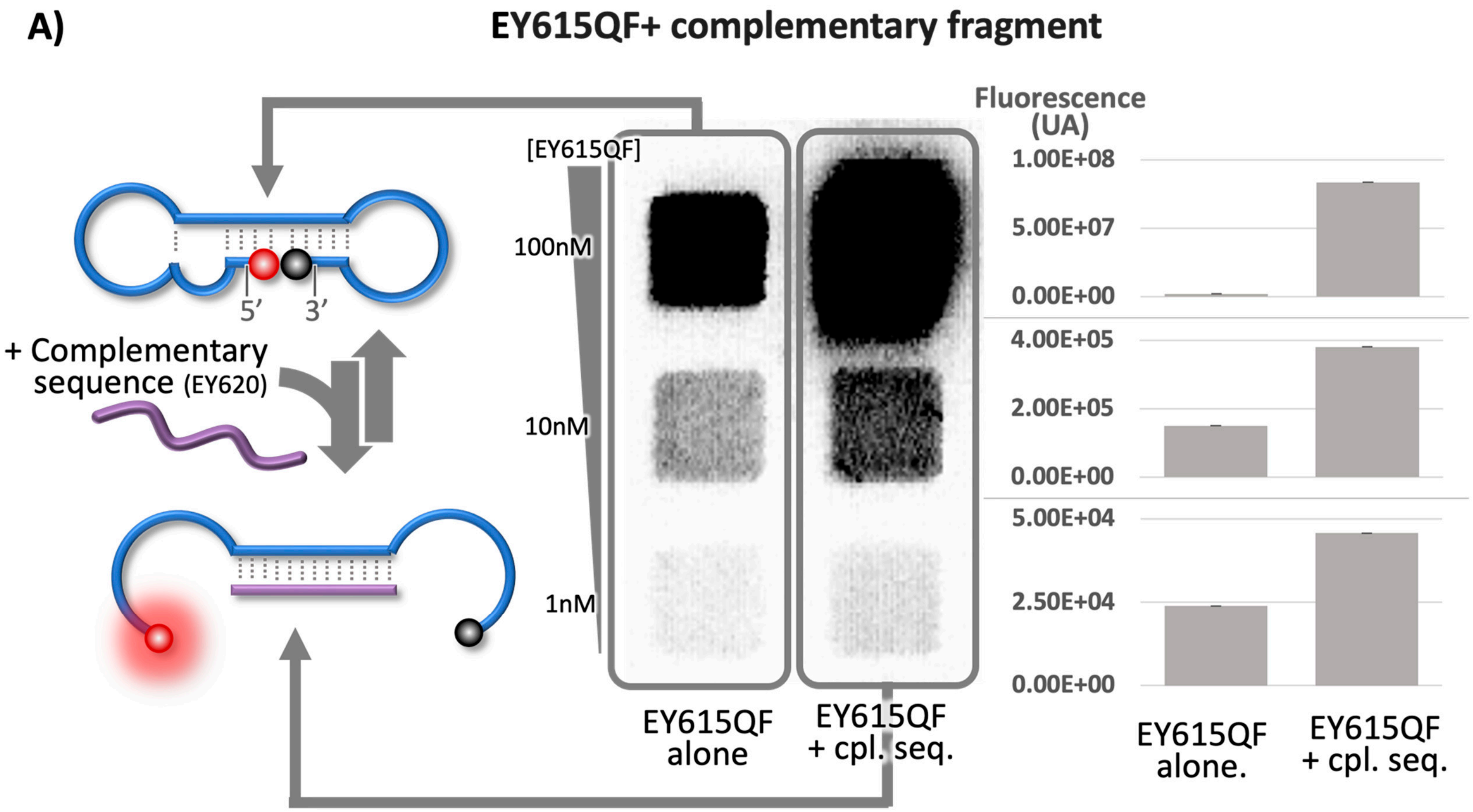

Figure S4: Fluorescence Quenching (EY615QF) and De-quenching (EY615QF + 10X complement) assessed with different concentrations of the EY615QF aptamer (sequence details in **Table S1**). Left section: anticipated fluorescence events, right section: fluorescence measurements**.**

References:

1. Markham, N. R. & Zuker, M. UNAFold: Software for nucleic acid folding and hybridization. *Methods in Molecular Biology* **453**, (2008).
2. Song, Y. *et al.* Discovery of Aptamers Targeting the Receptor-Binding Domain of the SARS-CoV-2 Spike Glycoprotein. *Anal Chem* **92**, (2020).
